# Dietary galacto-oligosaccharides prevent airway eosinophilia and hyperresponsiveness in a murine house dust mite-induced asthma model

**DOI:** 10.1186/s12931-015-0171-0

**Published:** 2015-02-07

**Authors:** Kim AT Verheijden, Linette EM Willemsen, Saskia Braber, Thea Leusink-Muis, Dianne JM Delsing, Johan Garssen, Aletta D Kraneveld, Gert Folkerts

**Affiliations:** Department of Pharmaceutical Sciences, Division of Pharmacology, Utrecht University, Faculty of Science, Utrecht, The Netherlands; Division of Veterinary Pharmacy, Pharmacology and Toxicology, Utrecht University, Faculty of Veterinary Sciences, Utrecht, The Netherlands; FrieslandCampina, Amersfoort, The Netherlands; Nutricia Research, Immunology, Utrecht, The Netherlands

**Keywords:** Asthma, House dust mite, Galacto-oligosaccharide, Budesonide

## Abstract

**Background:**

Allergic asthma is strongly associated with the exposure to house dust mite (HDM) and is characterized by eosinophilic pulmonary inflammation and airway hyperresponsiveness (AHR). Recently, there is an increased interest in using dietary oligosaccharides, also known as prebiotics, as a novel strategy to prevent the development of, or reduce, symptoms of allergy.

**Aim:**

We investigated the preventive capacity of dietary galacto-oligosaccharides (GOS) compared to an intra-airway therapeutic treatment with budesonide on the development of HDM-induced allergic asthma in mice.

**Methods:**

BALB/c mice were intranasally sensitized with 1 μg HDM on day 0 followed by daily intranasal challenge with PBS or 10 μg HDM on days 7 to 11. Two weeks prior to the first sensitization and throughout the experiment mice were fed a control diet or a diet containing 1% GOS. Reference mice were oropharyngeally instilled with budesonide (500 μg/kg) on days 7, 9, 11, and 13, while being fed the control diet. On day 14, AHR was measured by nebulizing increasing doses of methacholine into the airways. At the end of the experiment, bronchoalveolar lavage fluid (BALF) and lungs were collected.

**Results:**

Sensitization and challenge with HDM resulted in AHR. In contrast to budesonide, dietary intervention with 1% GOS prevented the development of AHR. HDM sensitization and challenge resulted in a significant increase in BALF leukocytes numbers, which was suppressed by budesonide treatment and dietary intervention with 1% GOS. Moreover, HDM sensitization and challenge resulted in significantly enhanced concentrations of IL-6, CCL17, IL-33, CCL5 and IL-13 in lung tissue. Both dietary intervention with 1% GOS or budesonide treatment significantly decreased the HDM-induced increased concentrations of CCL5 and IL-13 in lung tissue, while budesonide also reduced the HDM-enhanced concentrations of IL-6 and CCL17 in lung tissue.

**Conclusion:**

Not only did dietary intervention with 1% GOS during sensitization and challenge prevent the induction of airway eosinophilia and Th2-related cytokine and chemokine concentrations in the lung equally effective as budesonide treatment, it also prevented AHR development in HDM-allergic mice. GOS might be useful for the prevention and/or treatment of symptoms in asthmatic disease.

**Electronic supplementary material:**

The online version of this article (doi:10.1186/s12931-015-0171-0) contains supplementary material, which is available to authorized users.

## Introduction

Asthma is a complex disease from which the exact underlying immunological processes are still not fully understood [1]. According to the World Health Organization, 235 million people suffer from asthma worldwide and it is a common disease among children [2]. In asthma, the Th2 immune response leads to eosinophilic inflammation in the airways, mucus hypersecretion and airway hyperresponsiveness (AHR) [3]. During sensitization, an antigen activates the airway epithelial cells that in turn, via the release of several chemokines (e.g. CCL5) and cytokines (e.g. IL-33), results in the activation of innate immune cells such as dendritic cells (DC). Innate immune cells release Th2 polarizing cytokines, such as CCL17 and CCL22. In particular, high concentrations of IL-33 are expressed in epithelial cells of asthmatic patients [4,5]. Activated DC take up the allergenic protein and present their peptides to naïve T cells in draining lymph nodes. The naïve T cells develop into antigen-specific Th2 cells [6]. Upon subsequent antigen challenges, antigen-specific Th2 cells are activated to release IL-4, IL-5 and IL-13, resulting in the development of allergen-specific IgE producing plasma cells and generation and infiltration of eosinophils [7-11]. Allergen-specific IgE binds to mast cells in the airways and a second exposure to antigen results in the degranulation of mast cells. The influx of inflammatory cells (eosinophils, Th2 cells and mast cells) and production of mediators (TNF-a, IL-4, IL-5, IL-6, IL-13, IL-33) ultimately leads to acute bronchoconstriction, increased mucus production and AHR [11-13]. Currently, the treatment of asthma focuses on symptom relief only, using long-acting beta agonists with or without glucocorticosteroids, which is considered highly effective and safe. However, in many patients, the disease remains poorly controlled [14]. Long-term treatment with glucocorticosteroids can also have considerable side effects, such as weight gain, muscle weakness, reduced growth in children and osteoporosis in elderly [15]. In severe asthma patients long-term treatment can even induce glucocorticosteroid-resistance [16]. Therefore, novel preventive and/or therapeutic approaches are needed. Recent experiments have demonstrated a substantial influence of the gut microbiota on immune function beyond the gut. Development of asthma and allergies might even be due to the changes in gut microbiota [17-19]. Galacto-oligosaccharides (GOS) are non-digestible carbohydrates with prebiotic capacity, meaning that they selectively support growth and/or activity of bifidobacteria and lactobacilli. These bacteria are associated with a positive health benefit [17,20]. In addition, *in vivo, in vitro* as well as clinical research has shown benefits of GOS on the digestive and immune health [21-23]. Various animal studies have shown a preventive effect of non-digestible oligosaccharides on allergic diseases. In food allergic mice, a combination of GOS/long-chain fructo-oligosaccharides (lcFOS) with *Bifidobacterium breve* M-16 V was able to reduce allergic responses [24]. Van de Pol *et al.* used the same combination in patients with asthma and showed an increased peak expiratory flow, but no effect was seen on bronchial inflammation [25]. Vos *et al.* used a combination of GOS, lcFOS, and pectin-derived acidic oligosaccharides in an ovalbumine-induced asthma mouse model and showed a significant suppression of the airway inflammation and airway hyperreactivity [26]. In a murine OVA-induced chronic asthma model, Sagar *et al.* showed a decrease in pulmonary inflammation and airway remodeling after long-term treatment with scFOS/lcFOS/AOS in combination with *Bifidobacterium breve* [27]. Also treatment with *Bifidobacterium breve* alone was as effective as budesonide in reducing airway remodeling, but not in reducing lung resistance [28]. The development of allergic asthma is strongly associated with the exposure to house dust mite (HDM) [29]. For this reason, this study uses a HDM-induced allergic asthma model to study the preventive effect of dietary GOS on the AHR, pulmonary inflammation and lung cytokine concentrations in comparison with the therapeutic treatment budesonide.

## Materials and methods

### Mice

Male BALB/c mice (Charles River, Maastricht, The Netherlands), 6- to 8-week old (20–25 g), were used in all experiments. Mice were housed under bio-contained sterile conditions using HEPA® filtered isocages® (Tecniplast, Italy). Food and water were provided *ad libitum*. All animal experiments were conducted in compliance with the Guidelines of the Ethical Committee on the Use of Laboratory Animals of the Utrecht University (DEC 2013.II.01.003).

### HDM murine asthma model

**Figure 1:**
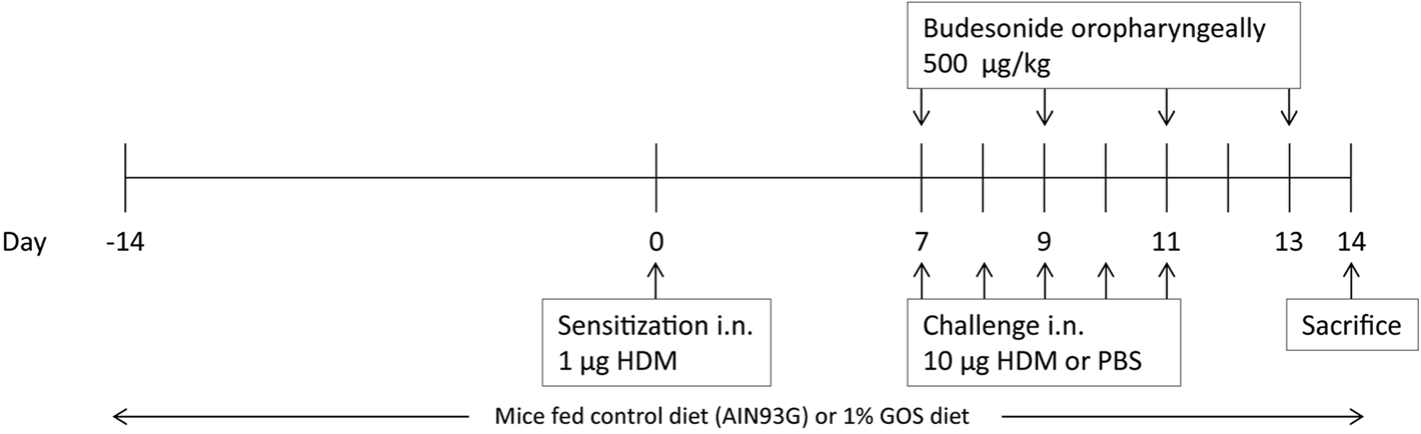
**Time schedule of the house dust mite allergic asthma model.** Male BALB/c mice were sensitized intranasally (i.n.) with house dust mite (HDM) on day 0 and were challenged on days 7 to 11 i.n. with HDM or PBS. Mice were fed control diet (AIN93G, contr) or 1% v/w GOS from day −14 to 14. In a separate set of animals, control mice and HDM-allergic mice were oropharyngeally instilled with budesonide on days 7, 9, 11 and 13. All mice were sacrificed on day 14.

While under isoflurane anaesthesia, BALB/c mice were intranasally (i.n.) sensitized with 1 μg HDM/40 μL PBS (Greer Laboratories, Lenoir, USA) on day 0 and challenged daily on days 7 to 11 with PBS (control, HDM-PBS) or 10 μg HDM/40 μL PBS (HDM-HDM) [30]. From day −14 to 14, mice were fed a control diet (AIN93G, contr) or the same diet containing 1% v/w GOS (Vivinal® GOS syrup with approximately 59% galacto-oligosaccharides, 21% lactose, 19% glucose, and 1% galactose on dry matter (dry matter of 75%); FrieslandCampina Domo, Borculo, The Netherlands). Carbohydrates in Vivinal® GOS were compensated isocalorically in the control diet by means of cellulose (for GOS), lactose (for lactose), and dextrose (for glucose). A separate control and HDM-allergic group were treated with budesonide as a reference treatment while being fed the control diet. After induction of a light isoflurane anaesthesia, mice were instilled oropharyngeally, with budesonide (500 μg/kg, Sigma-Aldrich, Zwijndrecht, The Netherlands) on days 7, 9 and 11, 6 h prior to the daily challenge and on day 13, 24 h prior to the assessment of airway responsiveness to methacholine (Figure 1) [28,31].

### Airway responsiveness measurement

Mice were intraperitoneally (i.p.) anaesthetized with a mix containing Ketamine (Vetoquinol S.A., Lure Cedex, France; 125 mg/kg) and Medetomidine (Pfizer, Capelle a/d Ijssel, Netherlands; 0.4 mg/kg). Lung function was assessed using EMKA invasive measurement of dynamic resistance (EMKA Technologies, Paris, France) in response to increasing doses of methacholine (acetyl-β-methyl-choline chloride, Sigma-Aldrich, Zwijndrecht, The Netherlands) (0–25 mg/mL, 10% puff for 10 sec.). Data are presented as average lung resistance (R_L_) in cm H_2_O/mL*sec^−1^ [28].

### Bronchoalveolar lavage

Mice were killed with an intraperitoneal overdose of pentobarbital (600 mg/kg, Nembutal™, Ceva Santé Animale, Naaldwijk, The Netherlands) after the airway responsiveness measurement. A small incision was made in the trachea to insert a cannula. Lungs were lavaged with 1 mL of pyrogen-free saline (0.9% NaCl, 37°C) supplemented with protease inhibitor cocktail tablet (Complete Mini, Roche Diagnostics, Mannheim, Germany). The supernatant of the first mL was used for cytokine and chemokine measurement. Afterwards, the lungs were lavaged 3 times with 1 mL saline solution (0.9% NaCl, 37°C). The BALF cells were centrifuged (400 × g, 5 min.) and pellets of the 4 lavages were pooled, resuspended and total numbers of BAL cells were counted using a Bürker-Türk chamber (magnification 100x). For differential BAL cell counts, cytospin preparations were made and stained with Diff-Quick (Merz & Dade A.G., Düdingen, Switzerland). Numbers of macrophages, lymphocytes, neutrophils and eosinophils were scored with light microscopy [32].

### Preparation of lung homogenates

In brief, lung samples were homogenized in 1% Triton X100 (Sigma-Aldrich)/PBS containing protease inhibitor (Complete Mini, Roche Diagnostics, Mannheim, Germany) using a Precellys 24 tissue homogenizer (Bertin Technologies, France) 3 times for 10 sec. at 6,000 rpm with a minimum of 5 min. cooling period on ice in between. Homogenates were centrifuged at 14,000 rpm for 5 min., supernatants collected and stored at −20°C until further use. The protein concentration of each sample was assayed using the Pierce BCA protein assay kit standardized to BSA according to the manufacturer's protocol (Thermo Fisher Scientific, Rockford, IL, USA). The homogenates were diluted to a final concentration of mg protein/mL [33,34].

### Measurement of cytokines

A standard Th1/Th2/Th17 assay (IL-2, −4, −6, −10, TNF-a, IFNγ; BD Biosciences, Breda, The Netherlands) was used to determine cytokine concentrations in lung homogenates according to the manufacturer’s instructions. Only the IL-6 concentration was assessed since all other cytokines concentrations were below the detection limit. IL-33, CCL5 and CCL17 were measured with a DuoSet ELISA (R&D Systems, Minneapolis, Minnesota, USA), IL-13 and IL-5 with a Ready-SET-Go!® ELISA (eBioscience, San Diego, CA, USA). The concentrations of these cytokines were expressed as pg/mg protein in lungs and pg/mL in BALF.

### Statistical analysis

Results are presented as mean ± standard error of mean (SEM). Data were statistically analyzed using a one-way ANOVA followed by a Bonferroni’s multiple comparisons test. P < 0.05 were considered significant. Statistical analyses were conducted using GraphPad Prism software (version 6.04).

## Results

### Airway hyperresponsiveness was abrogated upon dietary intervention with 1% GOS in HDM-allergic mice

**Figure 2:**
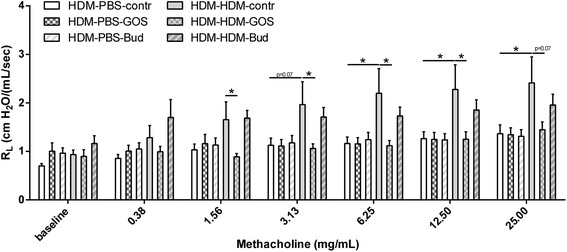
**Airway hyperresponsiveness was abrogated upon dietary intervention with 1% GOS in HDM allergic mice.** Airway resistance (R_L_) in response to increasing doses of methacholine on day 14. HDM-PBS: HDM-sensitized and PBS-challenged mice, HDM-HDM: HDM-sensitized and -challenged mice. Contr: control diet; GOS: 1% GOS diet; Bud: budesonide treatment. Results are shown as mean ± SEM. Statistical significance of differences was tested using post hoc Bonferroni’s multiple comparisons test after One-Way ANOVA. *P < 0.05, n = 7–8 mice/group.

Airway hyperresponsiveness (AHR) as a measure of lung function upon HDM exposure was determined in mice that were fed the control diet or the 1% GOS diet or those treated with budesonide. The basal airway responsiveness (0.70 ± 0.05 cm H_2_O/(mL/sec) in HDM-PBS-control group did not differ between the experimental groups. Moreover, an aerosol with saline (as a control for methacholine) did not change basal lung resistance (R_L_ 0.79 ± 0.06 cm H_2_O/(mL/sec) in HDM-PBS-contr group. HDM-HDM mice fed the control diet showed a significant increase in airway hyperresponsiveness (6.25-25 mg/mL of methacholine) compared to the HDM-PBS control group. Dietary intervention with 1% GOS resulted in a significant inhibition of AHR, reducing it back to control level, in HDM-allergic mice. Treatment with budesonide did not significantly affect AHR in HDM-HDM mice (Figure 2).

### Dietary intervention with 1% GOS reduced pulmonary eosinophilic inflammation in the lungs of HDM-allergic mice

**Figure 3:**
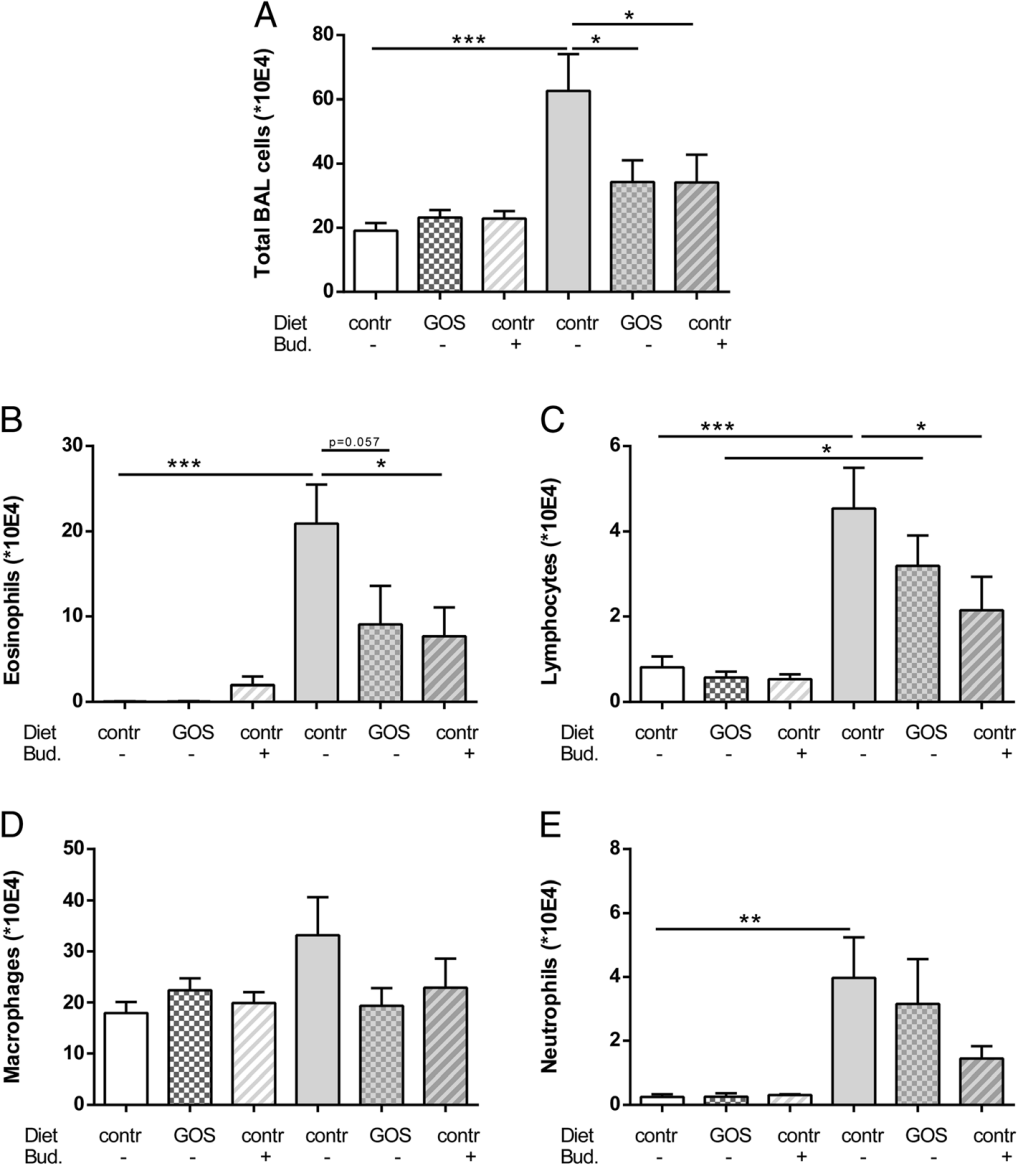
**Dietary intervention with 1% GOS reduced pulmonary eosinophilic inflammation in the lungs of HDM allergic mice.** Infiltration of inflammatory cells in the BALF of house dust mite allergic mice. HDM-PBS: HDM- sensitized and PBS-challenged mice (white bars), HDM-HDM: HDM-sensitized and -challenged mice (grey bar). Contr: control diet, GOS: 1% GOS diet, Bud: budesonide treatment. Total BAL cells **(A)**, absolute number of eosinophils **(B)**, lymphocytes **(C)**, macrophages **(D)** and neutrophils **(E)**. Results are shown as mean ± SEM. Statistical significance of differences was tested using post hoc Bonferroni’s multiple comparisons test after One-Way ANOVA. *P < 0.05, **P < 0.01, ***P < 0.001, n = 7–9 mice/group.

To investigate the inflammatory cell influx into the airways of HDM-allergic mice upon dietary intervention with 1% GOS or intra-airway treatment with budesonide, BALF was examined (Figure 3A). The total number of inflammatory cells in the BALF of HDM-HDM mice fed the control diet was significantly increased (Figure 3A), which was mainly due to an increase in the number of eosinophils and macrophages, and there was also significant increase in the number of lymphocytes and neutrophils (Figure 3B-E) compared to the HDM-PBS control group. Dietary intervention with 1% GOS reduced the total number of BALF cells and number of eosinophils (>57%, P = 0.05) in HDM-allergic mice (Figure 3B). Treatment with budesonide significantly reduced the total number of inflammatory cells in HDM-allergic mice compared to the HDM-HDM group fed the control diet (Figure 3A), which was due to a significant decrease in eosinophil and lymphocyte numbers (Figure 3B, E).

### The effect of 1% GOS on enhanced concentrations of IL-6, CCL17, IL-33, CCL5 and IL-13 in lungs of HDM-allergic mice

**Figure 4:**
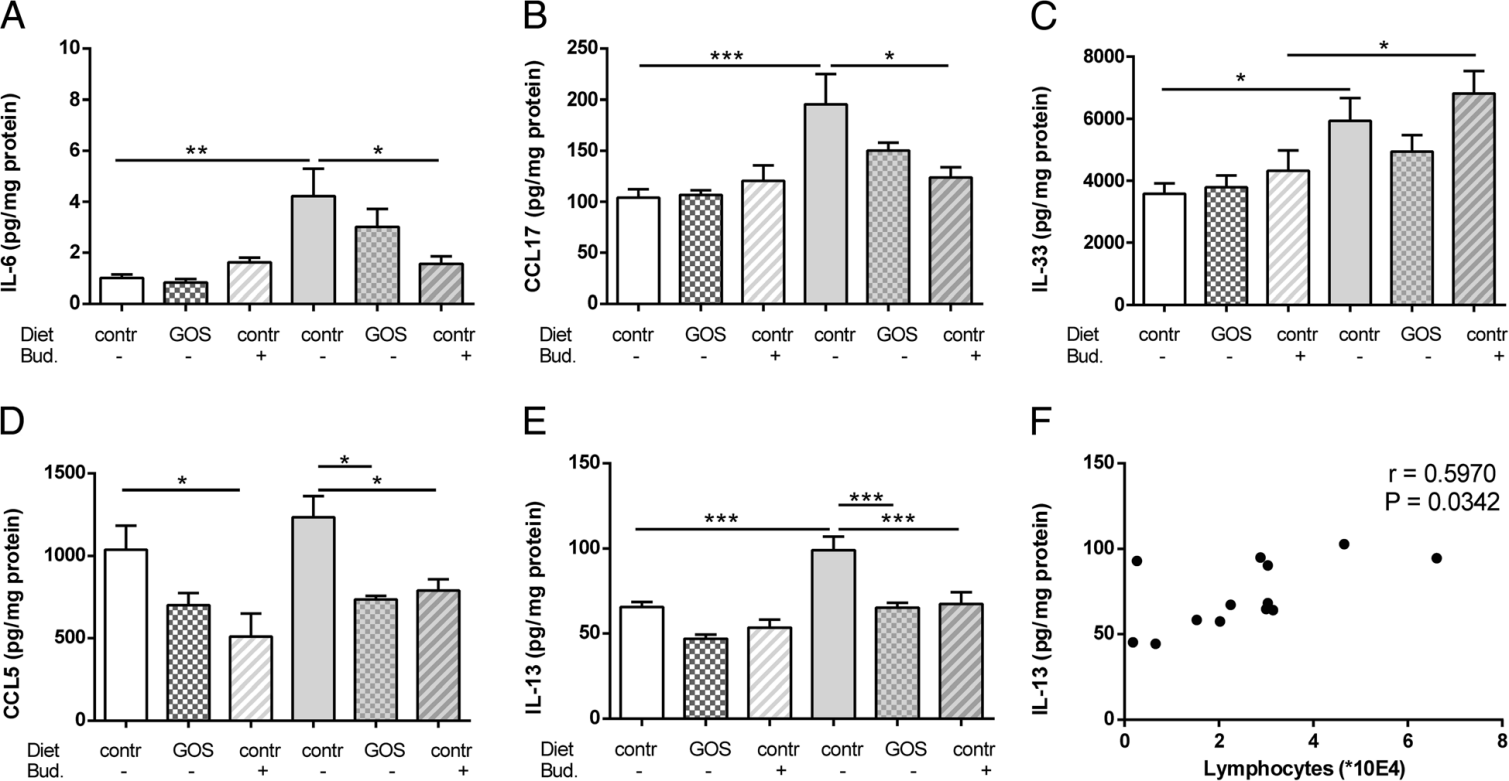
**IL-6, CCL17, IL-33, CCL5 and IL-13 concentrations in lungs of HDM allergic mice.** IL-6 **(A)**, CCL17 **(B)**, IL-33 **(C)**, CCL5 **(D),** and IL-13 **(E)** concentrations were measured in supernatant of lung homogenates. Correlation of IL-13 and the number of lymphocytes **(F)**. HDM-PBS: HDM-sensitized and PBS- challenged mice (white bars), HDM-HDM: HDM-sensitized and -challenged mice (grey bar). Contr: control diet, GOS: 1% GOS diet, Bud: budesonide treatment. Statistical significance of differences was tested using post hoc Bonferroni’s multiple comparisons test after One-Way ANOVA. *P < 0.05, **P < 0.01, ***P < 0.001, n = 6–8 mice/group. Correlation was analyzed using the Spearman correlation test.

In order to determine the effect of 1% GOS on pulmonary IL-6 concentrations, lungs were homogenized and IL-6 concentration was measured in the supernatant. The lung tissue concentration of IL-6 was significantly increased in HDM-HDM mice fed the control diet compared to the HDM-PBS control group. After dietary intervention with 1% GOS, IL-6 concentrations were not significantly changed in the lung tissue of HDM-allergic mice compared to controls. Treatment with budesonide of HDM-HDM mice significantly decreased the IL-6 concentration when compared with non-treated HDM-allergic mice (Figure 4A). In addition, CCL17 pulmonary concentrations were significantly increased in HDM-HDM mice fed the control diet compared to HDM-PBS control groups. Treatment with budesonide significantly decreased the CCL17 concentration when compared with non-treated HDM-allergic mice, 1% GOS showed a reduction of >20% (Figure 4B). Moreover, the IL-33 concentration was significantly increased in supernatants of HDM-HDM mice compared to the HDM-PBS control group. In the 1% GOS treated HDM-allergic mice, IL-33 was not changed while it was still enhanced in the budesonide-treated HDM-allergic group (Figure 4C). After dietary intervention with 1% GOS, CCL5 concentrations were significantly decreased in both HDM-allergic and control mice when compared to the non-treated groups, respectively. Treatment with budesonide also significantly decreased the CCL5 concentrations in the supernatant of lung tissue obtained from HDM-allergic and control mice. However, budesonide treatment already had an effect on CCL5 concentrations in HDM-PBS mice (Figure 4D). The concentration of theTh2 cytokine IL-13 in the lungs was significantly increased in HDM-HDM mice fed a control diet compared to the HDM-PBS control group. Both 1% GOS and budesonide significantly decreased the HDM allergy-induced increase in IL-13 (Figure 4E). Moreover, IL-13 concentrations in lung homogenates of HDM-HDM mice positively correlated with the number of lymphocytes (Figure 4F).

## Discussion

The aim of this study was to investigate the preventive effect of 1% dietary GOS on lung function and pulmonary inflammation in a murine model of HDM-induced allergic asthma. As is usually done for potentially new preventive and/or therapeutic agents, we compared the effectiveness with a golden standard reference treatment, the corticosteroid budesonide. To understand the underlying pathophysiology of the disease, animal models for allergic asthma are used. Here, a murine model for HDM-allergic asthma was used that mimics human features of asthmatic disease such as HDM-induced AHR, airway inflammation and pulmonary cytokine release [11,35,36]. Airway responsiveness upon methacholine exposure in HDM-allergic mice was significantly increased when compared to HDM-PBS control mice. Moreover, total inflammatory cell numbers were significantly increased in the BALF of HDM-allergic mice when compared to control mice. Budesonide treatment showed no significant decrease on the development of AHR whereas dietary intervention with 1% GOS prevented AHR development in HDM-allergic mice. However, both interventions suppressed airway inflammation in HDM-allergic mice. Both 1% GOS and budesonide were effective in the suppression of airway inflammation and decreased the number of eosinophils and macrophages. Furthermore, 1% GOS prevented the increase of CCL17 and IL-33 and significantly decreased CCL5 and IL-13 concentrations in the lungs. To our knowledge, this is the first study to demonstrate that dietary intervention with 1% GOS during sensitization and challenge is as effective as treatment with budesonide on allergic HDM asthma symptoms in a murine model. IL-6 concentrations have been reported to be increased in serum, BALF and sputum of asthmatic patients [12,37]. This increase was also observed in the murine model of HDM-induced allergic asthma. Budesonide, but not 1% GOS, normalized HDM allergy-induced increase of IL-6 concentrations to the control level. This effect of budesonide is in agreement with clinical studies which demonstrate that glucocorticosteroids decrease IL-6 concentrations in BALF in asthmatic patients in association with decreased activation and recruitment of inflammatory cells in asthma [38]. In our study, budesonide decreased the number of inflammatory cells as well. Besides being released by macrophages and T cells, IL-6 is highly expressed by epithelial cells obtained from allergic asthma patients [39]. However, the exact role of IL-6 in asthma pathology is not fully understood and has to be further elucidated. In patients with asthma, CCL17 has been found in increased concentrations in serum and BALF [40]. Previously, an important role for CCR4 and its ligand CCL17 in Th2 T cell recruitment has been demonstrated in asthma [41]. Budesonide significantly decreased the HDM allergy-induced levels of CCL17, whereas 1% GOS showed a reduction of >20%. The latter is in agreement with studies from Leung *et al.*, who found higher concentrations of CCL17 in the serum of non-steroid-treated asthmatic children compared with steroid-treated asthmatic children [42]. IL-33 is known to contribute to AHR since animal studies demonstrated that intranasally administered IL-33 results in an AHR-association with eosinophilia, goblet cell hyperplasia, and accumulation of IL-4, −5 and −13 in the lungs [43,44]. IL-33 is produced by epithelial cells after allergen stimulation, is a chemoattractant for Th2 cells and can activate mast cells to release CXCL8, IL-5, −6, and −13 [45]. Indeed, IL-33 may be involved in human asthmatic disease, since it is increased in BALF of moderate asthma patients as compared to mild asthma patients or controls [46]. Typically the concentration of IL-33 in the lung tissue obtained in our study was significantly enhanced in HDM-allergic and budesonide-treated mice compared to the control mice. In these budesonide-treated mice, the AHR was still evident. Indeed, Deckers *et al*. also demonstrated that budesonide had no effect on IL-33 concentrations in asthmatic patients [47]. However in HDM-allergic mice fed 1% GOS the HDM induced increase in IL-33 in lung tissue was prevented. Since IL-33 is one of the factors contributing to AHR [43,44], this could relate to the 1% GOS-induced abrogation of the AHR response of these mice. CCL5 is produced at high concentrations within the airway epithelium of human asthmatics and in turn will target eosinophils to the airways [48]. In this study, both dietary 1% GOS as well as budesonide treatment showed a significant decrease in CCL5 concentrations in lung tissue of HDM-allergic mice. Dampening of pulmonary CCL5 concentrations could be the mechanism by which GOS and budesonide treatment induces an abrogation of eosinophil infiltration in the lungs of HDM-allergic mice. In humans, the release of the Th2 cytokine IL-5 leads to activation of the eosinophil/basophil lineage. Increased eosinophil cell influx and AHR are strongly associated in asthmatic patients and can be provoked after inhalation of IL-5. However, there are many studies in humans demonstrating that treatment with anti-IL-5- specific antibodies reduced the number of eosinophils in sputum and blood of mild and severe asthmatic patients, although the AHR was not affected [49]. Thus, inflammatory pathways underlying IL-5 alone are not sufficient for the development of AHR in allergic asthma. In our study, IL-5 tended to be increased in the BALF of HDM-allergic mice whereas this did not occur after dietary intervention with 1% GOS or treatment with budesonide (Additional file 1: Figure S1). Indeed, IL-5 in BALF was positively correlated with eosinophil numbers (Additional file 1: Figure S1). The concentration of another Th2 cytokine, IL-13, in lung tissue was significantly increased in HDM-allergic mice compared to the control mice. These data are in agreement with previous studies that show a central role for IL-13 in generating the murine allergic AHR following sensitization and challenge of HDM [50]. Many of the processes involved in allergic asthma can be directed to IL-13. Besides being secreted by Th2 cells, IL-13 is also secreted by mast cells and innate lymphoid cells. In the current study, IL-13 concentrations in lung tissue were positively correlated with BALF lymphocyte numbers, suggesting this subset to be an important source of IL-13. IL-13 also triggers macrophage and eosinophil activation which, in turn, can contribute to AHR [51]. Furthermore, IL-13 is increased in BALF and bronchial biopsy specimens of asthmatic patients and known to be inhibited by glucocorticoids [51,52]. Both dietary 1% GOS as well as budesonide showed a significant decrease in IL-13 concentrations in lung homogenates of HDM-allergic mice, hereby dampening a major contributor to asthmatic inflammation. In previous studies*, Bifidobacterium breve* or *Lactobacillus rhamnosus,* either or not combined with specific oligosaccharides, suppressed airway inflammation in a murine model for OVA induced chronic asthma [27,28]. Our studies show similar effects of only GOS in an acute model for HDM induced asthma. As shown in earlier studies with dietary oligosaccharides, it is known that they have a positive effect on the composition of microbiota [21-23]. A potential mechanism of GOS could be that by changing the microbiota, immunomodulation via intestinal epithelial signaling occurs leading to systemic effects resulting in a decreased HDM immune response, as has been suggested by several studies [17,20]. In conclusion, in our study budesonide suppressed inflammatory cell numbers and cytokine concentrations of IL-6, CCL17, CCL5 and IL-13 in HDM allergic mice. However, budesonide did not modulate the HDM-allergy induced AHR and increased the pulmonary tissue concentrations of IL-33. Interestingly, dietary intervention with 1% GOS prevented the development of AHR and suppressed airway eosinophilia in HDM allergic mice. Moreover, 1% GOS prevented the increase in IL-33 and abrogated the HDM-induced CCL17, CCL5 and IL-13 release in the lungs of HDM-allergic mice. Dietary intervention with 1% GOS may be beneficial in the prevention of HDM-induced allergic asthma, and may offer a potential novel strategy with less side effects than current therapeutic treatments. However, more research is needed to demonstrate this beneficial effect. In addition, the mechanism of the immune modulating functions of 1% GOS needs to be further elucidated, as well as the most effective dose.

## Additional file

Additional file 1:
**Measurement of IL-5 in BALF.** IL-5 concentrations were measured with a Ready-SET-Go!® ELISA (eBioscience, San Diego, CA, USA). The concentration of this cytokine was expressed as pg/mL. **Figure S1.** IL-5 concentrations in the BALF of HDM allergic mice. IL-5 concentration (A) was measured in the BALF. Correlation of IL-5 and the number of eosinophils (B). HDM-PBS: HDM-sensitized and PBS-challenged mice (white bars), HDM-HDM: HDM-sensitized and -challenged mice (grey bar). Contr: control diet, GOS: 1% GOS diet, Bud: budesonide treatment. Statistical significance of differences was tested using post hoc Bonferroni’s multiple comparisons test after One-Way ANOVA. Correlation was analyzed using the Spearman correlation test.

## Abbreviations

AHR: Airway Hyper Responsiveness
BALF: Broncho-Alveolar Lavage Fluid
GOS: Galacto-oligosaccharide
HDM: House Dust Mite
R_L_: Lung Resistance
