# Supplementary figures and images for: Dietary galacto-oligosaccharides prevent airway eosinophilia and hyperresponsiveness in a murine house dust mite-induced asthma model
Source: Respir Res. 2015 Feb 7;16(1):17. doi: 10.1186/s12931-015-0171-0 (PMC4327967; doi:10.1186/s12931-015-0171-0)

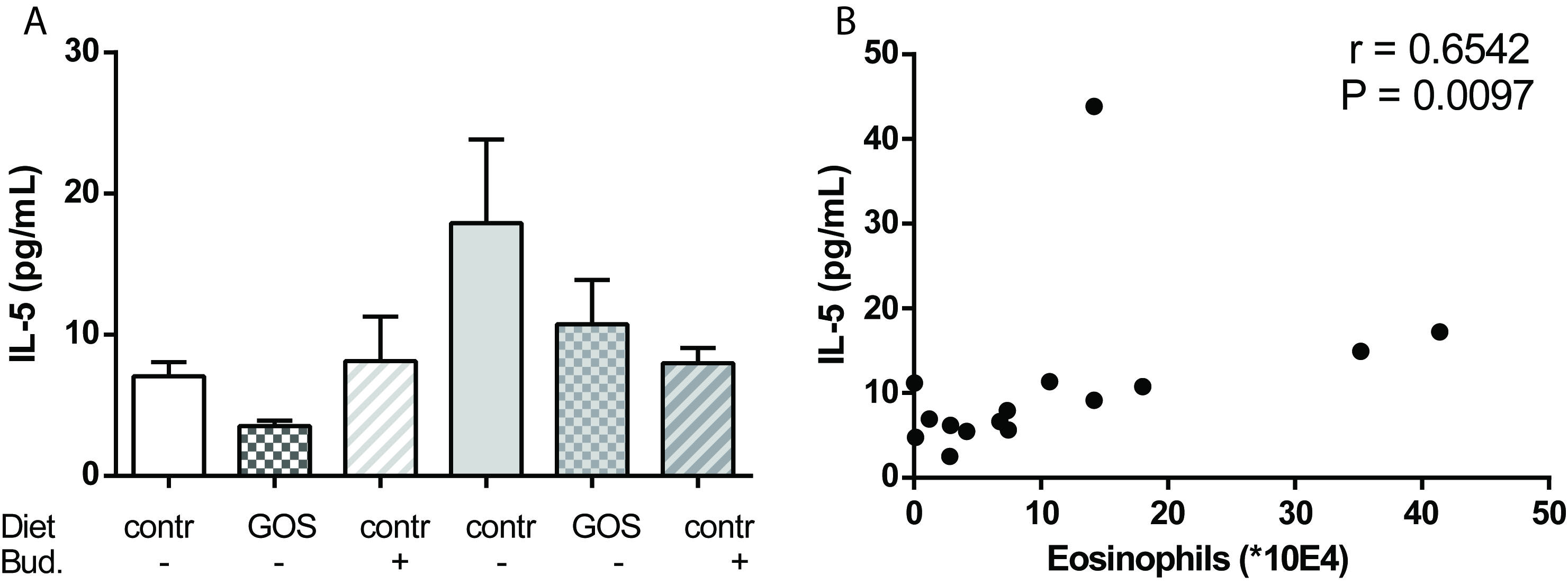

Supplement: Additional file 1: — Measurement of IL-5 in BALF. IL-5 concentrations were measured with a Ready-SET-Go!® ELISA (eBioscience, San Diego, CA, USA). The concentration of this cytokine was expressed as pg/mL. Figure S1. IL-5 concentrations in the BALF of HDM allergic mice. IL-5 concentration (A) was measured in the BALF. Correlation of IL-5 and the number of eosinophils (B). HDM-PBS: HDM-sensitized and PBS-challenged mice (white bars), HDM-HDM: HDM-sensitized and -challenged mice (grey bar). Contr: control diet, GOS: 1% GOS diet, Bud: budesonide treatment. Statistical significance of differences was tested using post hoc Bonferroni’s multiple comparisons test after One-Way ANOVA. Correlation was analyzed using the Spearman correlation test. [file 12931_2015_171_MOESM1_ESM.tiff]
